# COVID-19–Associated Hospitalizations Among U.S. Adults Aged ≥18 Years — COVID-NET, 12 States, October 2023–April 2024

**DOI:** 10.15585/mmwr.mm7339a2

**Published:** 2024-10-03

**Authors:** Christopher A. Taylor, Kadam Patel, Huong Pham, Pam Daily Kirley, Breanna Kawasaki, James Meek, Lucy Witt, Patricia A. Ryan, Libby Reeg, Kathy Como-Sabetti, Adrienne Domen, Bridget Anderson, Sophrena Bushey, Melissa Sutton, H. Keipp Talbot, Emma Mendez, Fiona P. Havers, COVID-NET Surveillance Team, COVID-NET Surveillance Team, Jeremey Roland, Nisha Alden, Daewi Kim, Kyle P. Openo, Maya L. Monroe, Val Tellez Nunez, Erica Bye, Dominic Solhtalab, Grant Barney, Christina B. Felsen, Nasreen Abdullah, William Schaffner, Isabella Reyes

**Affiliations:** ^1^Coronavirus and Other Respiratory Viruses Division, National Center for Immunization and Respiratory Diseases, CDC; ^2^California Emerging Infections Program, Oakland, California; ^3^Colorado Department of Public Health & Environment; ^4^Connecticut Emerging Infections Program, Yale School of Public Health, New Haven, Connecticut; ^5^Division of Infectious Diseases, Emory University School of Medicine, Atlanta, Georgia; ^6^Georgia Emerging Infections Program, Atlanta, Georgia; ^7^Maryland Department of Health; ^8^Michigan Department of Health and Human Services; ^9^Minnesota Department of Health; ^10^University of New Mexico Emerging Infections Program, Albuquerque, New Mexico; ^11^New York State Department of Health; ^12^University of Rochester School of Medicine and Dentistry, Rochester, New York; ^13^Public Health Division, Oregon Health Authority; ^14^Vanderbilt University Medical Center, Nashville, Tennessee; ^15^Salt Lake County Health Department, Salt Lake City, Utah.; California Emerging Infections Program; Colorado Department of Public Health & Environment; Connecticut Emerging Infections Program; Emory University School of Medicine, Georgia Emerging Infections Program, Department of Public Health, Atlanta Veterans Affairs Medical Center; Maryland Department of Health; Michigan Department of Health and Human Services; Minnesota Department of Health; University of New Mexico Emerging Infections Program; New York State Department of Health; University of Rochester School of Medicine and Dentistry; Public Health Division, Oregon Health Authority; Vanderbilt University Medical Center; Salt Lake County Health Department.

**Summary**

**What is already known about this topic?**

Hospitalization due to COVID-19 remains a public health concern. The risk for hospitalization among adults increases with age.

**What is added by this report?**

During October 2023–April 2024, adults aged ≥65 years accounted for 70% of all COVID-19–associated hospitalizations among adults. Most hospitalized adults had multiple underlying medical conditions. Only 12% had received the recommended COVID-19 2023–2024 formula vaccine.

**What are the implications for public health practice?**

Adults at increased risk for COVID-19–associated hospitalization should reduce their risk for severe COVID-19 by adopting measures to reduce risk for contracting COVID-19, receiving recommended COVID-19 vaccinations, and seeking prompt outpatient antiviral treatment after a positive SARS-CoV-2 test result.

## Abstract

Among adults, COVID-19 hospitalization rates increase with age. Data from the COVID-19–Associated Hospitalization Surveillance Network were analyzed to estimate population-based COVID-19–associated hospitalization rates during October 2023–April 2024 and identify demographic and clinical characteristics of adults aged ≥18 years hospitalized with COVID-19. Adults aged ≥65 years accounted for 70% of all adult COVID-19–associated hospitalizations, and their COVID-19–associated hospitalization rates were higher than those among younger adult age groups. Cumulative rates of COVID-19–associated hospitalization during October 2023–April 2024 were the lowest for all adult age groups during an October–April surveillance period since 2020–2021. However, hospitalization rates among all adults aged ≥75 years approached one COVID-19–associated hospitalization for every 100 persons. Among adults hospitalized with COVID-19, 88.1% had not received the 2023–2024 formula COVID-19 vaccine before hospitalization, 80.0% had multiple underlying medical conditions, and 16.6% were residents of long-term care facilities (LTCFs). Guidance for adults at high risk for severe COVID-19 illness, including adults aged ≥65 years and residents of LTCFs, should continue to focus on adopting measures to reduce risk for contracting COVID-19, advocating for receipt of recommended COVID-19 vaccinations, and seeking prompt outpatient antiviral treatment after receipt of a positive SARS-CoV-2 test result.

## Introduction

Hospitalization due to COVID-19 has remained a public health concern since the start of the COVID-19 pandemic. Persons of all ages remain at risk for COVID-19–associated hospitalization; among adults, the risk for hospitalization increases with age (*1*). Understanding the characteristics of adults hospitalized with COVID-19 can help guide appropriate recommendations as circulating SARS-CoV-2 variants change and vaccine recommendations are updated. Data from the COVID-19–Associated Hospitalization Surveillance Network (COVID-NET) were analyzed to estimate COVID-19–associated hospitalization rates during October 2023–April 2024 and identify demographic and clinical characteristics of adults aged ≥18 years hospitalized with COVID-19.

## Methods

### Data Source

COVID-NET conducts population-based surveillance for laboratory-confirmed COVID-19–associated hospitalization* among residents of predefined surveillance catchment areas. Demographic data were collected on all COVID-19–associated hospitalizations in 90 counties across 12 states^†^ and were used to calculate overall, age-stratified, and age-adjusted hospitalization rates from October 1, 2023, through April 27, 2024,^§^ and compare these rates with those from previous surveillance periods.^¶^

### Selection of Cases for Analysis

Using previously described methods (*2*), trained surveillance officers abstracted demographic and clinical data from the medical records of an age- and site-stratified monthly random sample of patients hospitalized during October 2023–April 2024. Analyses of sampled cases were limited to hospitalizations for which COVID-19–related illness was the likely primary complaint at the time of admission, based on information in the medical record (*3*). Data on receipt of the most recent patient COVID-19 vaccination** was obtained from state immunization information systems. Underlying conditions were defined as chronic or preexisting medical conditions present before or at the time of hospital admission. Long-term care facility (LTCF) residency was ascertained based on status upon admission.

### Data Analysis

For sampled data, unweighted case counts and weighted percentages that better represent the hospitalized population of the catchment area are presented (*2*). Data were analyzed using SAS (version 9.4; SAS Institute); variances were estimated using the Taylor series linearization method. This activity was reviewed by CDC, deemed not research, and was conducted consistent with applicable federal law and CDC policy.^††^

## Results

### Age Distribution of Hospitalized Adults with COVID-19

During October 2023–April 2024, COVID-NET identified 40,761 COVID-19–associated hospitalizations, 38,900 (95.4%) of which were among adults aged ≥18 years.^§§^ Among hospitalized adults, those aged 18–49, 50–64, 65–74, and ≥75 years accounted for 13.5%, 16.7%, 21.3%, and 48.6% (unweighted) of cases, respectively. Weekly proportions of adults with COVID-19–associated hospitalizations by age group have changed over time but were stable for this analytic period (Supplementary Figure 1, https://stacks.cdc.gov/view/cdc/162446).

### Cumulative Age- and Season-Specific COVID-19 Hospitalization Rates

**FIGURE 1:**
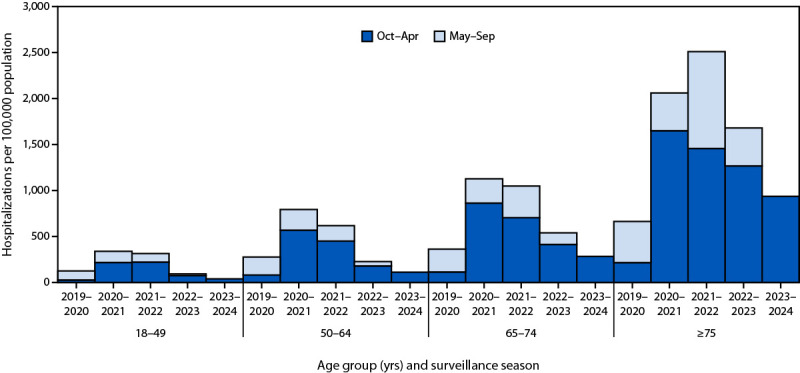
Cumulative* COVID-19–associated hospitalization**^†^** rates among adults aged ≥18 years, by age group and surveillance season**^§^** — COVID-19–Associated Hospitalization Surveillance Network, 12 states,**^¶^** March 2020–April 2024 * Cumulative rates are the sequential sum of weekly hospitalizations divided by the catchment area population. ^†^ COVID-19–associated hospitalizations among patients who received a positive SARS-CoV-2 test result during hospitalization or ≤14 days before admission. ^§^ The COVID-19–Associated Hospitalization Surveillance Network surveillance season extends year-round from epidemiologic week 40 through epidemiologic week 39, which roughly aligns with October–September of the following year. To compare with the analytic period in this study, the season was divided into surveillance weeks 40–17 (approximately October–April) and epidemiologic weeks 18–39 (approximately May–September). The 2019–2020 surveillance season began on March 1, 2020; data for that surveillance season are presented as epidemiologic weeks 10–17 and weeks 18–39. ^¶^ Selected counties and county equivalents in California, Colorado, Connecticut, Georgia, Maryland, Michigan, Minnesota, New Mexico, New York, Oregon, Tennessee, and Utah.

During October 2023–April 2024, cumulative COVID-19–associated hospitalization rates (hospitalizations per 100,000 population) among each adult age group were the lowest experienced during the months of October–April since the 2020–2021 surveillance season (Figure 1). Since 2020–2021, approximately 25% of COVID-19–associated hospitalizations among adults have occurred during May–September. During October 2023–April 2024, cumulative rates were highest among adults aged ≥75 years (936.4), approaching one COVID-19–associated hospitalization for every 100 persons. The rate in this group was also higher than that of any other age group during any previous October–April period. Relative to adults aged 18–49 years, cumulative rates among adults aged 50–64, 65–74, and ≥75 years during October 2023–April 2024 were 2.9, 7.3, and 24.1 times as high, respectively.

During October 2023–April 2024, weekly COVID-19–associated hospitalization rates increased during November–December, peaking in late December or early January, depending on the age group (Supplementary Figure 2, https://stacks.cdc.gov/view/cdc/162446). The peak weekly rate among adults aged ≥75 years (58.9) was 24.5 times as high as that among adults aged 18–49 years (2.4).

### COVID-19 Hospitalization Rates Among Racial and Ethnic Groups

**FIGURE 2:**
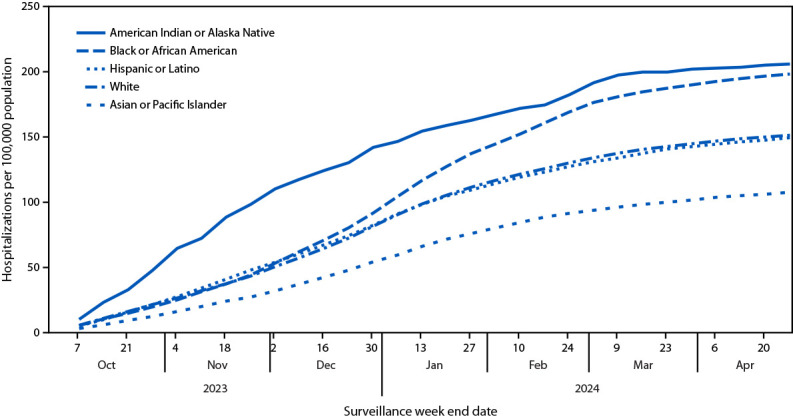
Cumulative* age-adjusted COVID-19–associated hospitalization**^†^** rates among adults aged ≥18 years, by race and ethnicity**^§^** — COVID-19–Associated Hospitalization Surveillance Network, 12 states,**^¶^** October 1, 2023–April 27, 2024 * Cumulative rates are the sequential sum of weekly hospitalizations divided by the catchment area population. ^†^ COVID-19–associated hospitalizations among patients who received a positive SARS-CoV-2 test result during hospitalization or ≤14 days before admission. ^§^ Persons of Hispanic or Latino (Hispanic) origin might be of any race but are categorized as Hispanic; all racial groups are non-Hispanic. ^¶^ Selected counties and county equivalents in California, Colorado, Connecticut, Georgia, Maryland, Michigan, Minnesota, New Mexico, New York, Oregon, Tennessee, and Utah.

During the same period, cumulative, age-adjusted COVID-19–associated hospitalization rates were highest among non-Hispanic American Indian or Alaska Native (AI/AN) (205.9) and non-Hispanic Black or African American (Black) (198.2) adults (Figure 2); rates among both groups were 1.4 times as high as rates among Hispanic or Latino (Hispanic) adults (149.5) and 1.3 times as high as rates among non-Hispanic White (White) adults (151.4). Whereas AI/AN adults experienced the highest hospitalization rates throughout the season, rates among Black adults increased more sharply during December 2023–February 2024 relative to other groups, leading to cumulative rates that were similar to those among AI/AN adults.

### Vaccination Status, Underlying Conditions, and Clinical Course Among Adults Hospitalized with COVID-19

Among a sample of 1,320 hospitalized adults,^¶¶^ 88.1% had not received the 2023–2024 formula COVID-19 vaccine dose (Table). In addition, 57.7% had not received the 2022–2023 formula (bivalent) dose, including 66.7% of those aged 65–74 years, and 46.5% of those aged ≥75 years, representing 52.5% (95% CI = 46.7%–58.2%) of adults aged ≥65 years.

**TABLE:** Demographic characteristics of and clinical outcomes among a sample of adults aged ≥18 years hospitalized with laboratory-confirmed SARS-CoV-2 infection,* by age group — COVID-19–Associated Hospitalization Surveillance Network, 12 states,^†^ October 2023–April 2024

Characteristic	Age group, yrs									
	Total		18–49		50–64		65–74		≥75	
	No.	Weighted % (95% CI)	No.	Weighted % (95% CI)	No.	Weighted % (95% CI)	No.	Weighted % (95% CI)	No.	Weighted % (95% CI)
**Total**	**1,320**	**100 (100.0–100.0)**	**338**	**100 (100.0–100.0)**	**485**	**100 (100.0–100.0)**	**159**	**100 (100.0–100.0)**	**338**	**100 (100.0–100.0)**
**Sex**										
Female	**716**	**51.2 (46.7–55.6)**	192	54.8 (46.9–62.6)	250	46.9 (39.9–54.0)	83	45.3 (35.2–55.7)	191	54.5 (47.4–61.4)
Male	**604**	**48.8 (44.4–53.3)**	146	45.2 (37.4–53.1)	235	53.1 (46.0–60.1)	76	54.7 (44.3–64.8)	147	45.5 (38.6–52.6)
**Race and ethnicity^§^**										
A/PI	**54**	**5.3 (3.3–7.9)**	12	4.1 (1.7–8.1)	22	5.9 (3.1–10.0)	—^¶^	—^¶^	13	5.0 (2.3–9.4)
AI/AN	**17**	**1.1 (0.5–2.3)**	—^¶^	—^¶^	—^¶^	—^¶^	—^¶^	—^¶^	—^¶^	—^¶^
Black or African American	**270**	**19.8 (16.4–23.5)**	94	33.4 (26.1–41.4)	117	31.7 (25.1–39.0)	27	20.1 (12.9–29.1)	32	13.0 (8.2–19.3)
White	**818**	**64.5 (60.1–68.6)**	162	39.0 (31.7–46.7)	276	47.8 (40.8–54.9)	110	64.4 (53.9–74.0)	270	74.9 (68.0–81.0)
Hispanic or Latino	**133**	**7.3 (5.5–9.6)**	54	18.2 (12.4–25.2)	54	11.7 (7.9–16.7)	10	6.8 (2.6–14.0)	15	4.1 (2.0–7.4)
Unknown race	**19**	**1.1 (0.4–2.5)**	—^¶^	—^¶^	—^¶^	—^¶^	—^¶^	—^¶^	—^¶^	—^¶^
**Resident of long-term care facility**										
Yes	**171**	**16.6 (13.4–20.2)**	24	7.8 (4.0–13.4)	48	11.8 (7.3–17.6)	14	7.7 (3.4–14.4)	85	23.7 (18.1–30.0)
No	**1,148**	**83.4 (79.8–86.6)**	314	92.2 (86.6–96.0)	436	88.2 (82.4–92.7)	145	92.3 (85.6–96.6)	253	76.3 (70.0–81.9)
**Underlying medical conditions**										
0	**95**	**3.5 (2.5–4.9)**	47	12.1 (7.9–17.5)	36	5.1 (3.1–7.8)	—^¶^	—^¶^	—^¶^	—^¶^
1	**254**	**16.5 (13.5–19.9)**	99	29.2 (22.7–36.4)	74	16.0 (11.2–21.8)	30	21.7 (13.2–32.5)	51	12.1 (8.4–16.8)
≥1	**1,225**	**96.5 (95.1–97.5)**	291	87.9 (82.5–92.1)	449	94.9 (92.2–96.9)	155	98.1 (94.6–99.6)	330	97.8 (95.2–99.2)
≥2	**971**	**80.0 (76.4–83.2)**	192	58.7 (50.9–66.1)	375	78.9 (72.9–84.1)	125	76.4 (65.7–85.1)	279	85.7 (80.8–89.8)
**Hospitalization, intervention or outcome**										
Length of stay, days, median (IQR)	**3.4 (1.9–7.1)**		2.9 (1.4–5.5)		3.4 (1.9–7.9)		3.2 (1.8–6.8)		3.6 (2.0–7.2)	
ICU admission	**247**	**18.4 (15.0–22.1)**	64	17.9 (12.6–24.3)	99	21.5 (16.0–28.0)	36	21.4 (13.7–31.0)	48	16.0 (11.1–22.1)
Invasive mechanical ventilation	**95**	**8.4 (5.9–11.6)**	21	5.9 (3.1–10.2)	40	11.3 (7.0–17.0)	21	12.8 (6.9–21.0)	13	6.1 (2.6–11.8)
In-hospital death	**60**	**6.9 (4.6–9.9)**	—^¶^	—^¶^	20	6.4 (3.0–11.7)	16	11.3 (5.6–19.8)	17	6.1 (3.0–11.0)
Any respiratory viral codetection**										
Yes	**47**	**4.4 (2.7–6.8)**	13	4.0 (1.6–8.2)	16	5.2 (2.0–10.7)	—^¶^	—^¶^	10	4.1 (1.7–8.1)
**Vaccination status^††^**										
No record of 2022–2023 (bivalent) or 2023–2024 formula dose	**766**	**57.7 (53.3–62.1)**	236	75.0 (68.1–81.0)	295	70.3 (64.0–76.1)	92	66.7 (57.0–75.5)	143	46.5 (39.5–53.6)
Received 2022–2023 (bivalent) dose, but no 2023–2024 formula dose	**401**	**30.3 (26.4–34.5)**	81	20.8 (15.2–27.3)	150	24.2 (19.0–30.0)	44	23.8 (16.5–32.6)	126	36.9 (30.4–43.7)
Received 2023–2024 formula dose	**146**	**11.9 (9.2–15.2)**	20	4.3 (2.1–7.7)	35	5.5 (2.7–9.7)	22	9.5 (4.6–16.8)	69	16.6 (11.9–22.2)
Did not receive 2023–2024 formula dose	**1,167**	**88.1 (84.8–90.8)**	317	95.7 (92.3–97.9)	445	94.5 (90.3–97.3)	136	90.5 (83.2–95.4)	269	83.4 (77.8–88.1)

**Abbreviations:** A/PI = Asian or Pacific Islander; AI/AN = American Indian or Alaska Native; ICU = intensive care unit.

* The likely primary complaint upon admission is identified by trained COVID-19–Associated Hospitalization Surveillance Network surveillance officers using information in the medical record. The likely primary complaint is identified and categorized as COVID-19–related illness; inpatient surgery or procedures; psychiatric admission requiring acute medical care; trauma; “other” (with an accompanying free-text field); or unknown. CDC clinicians independently review the free-text field of complaints classified as “other” to determine if the complaint might be recategorized or remain in the “other” category (e.g., skin and soft tissue infections). Hospitalizations for which the likely primary complaint was not COVID-19–related illness were excluded from the analysis of clinical data.

^†^ Selected counties and county equivalents in California, Colorado, Connecticut, Georgia, Maryland, Michigan, Minnesota, New Mexico, New York, Oregon, Tennessee, and Utah.

^§^ Persons of Hispanic or Latino (Hispanic) origin might be of any race but are categorized as Hispanic; all racial groups are non-Hispanic. Non-Hispanic persons of other races not listed are not presented due to small sample size.

^¶^ Data are not presented for cells with sample size <10.

** Denominators are the number of adults tested for respiratory viral codetections (1,134), accounting for 86.0% (95% CI = 82.5%–89.0%) of adults aged ≥18 years.

^††^ Vaccination status for the 2023–2024 surveillance season was only collected for vaccines administered on or after September 1, 2022.

Among this sample of adults hospitalized with COVID-19, 80.0% had at least two underlying medical conditions, and 16.6% were residents of LTCFs. In addition, 18.4% were admitted to an intensive care unit, 8.4% received invasive mechanical ventilation, and 6.9% died during hospitalization. Among all in-hospital deaths, 45.0% (95% CI = 26.0%–65.0%) were among persons aged ≥75 years.

## Discussion

During October 2023–April 2024, cumulative COVID-19–associated hospitalization rates were lower than those during previous years. Similar to previous surveillance seasons, adults aged ≥65 years experienced COVID-19–associated hospitalization rates many times higher than did adults in other age groups (*3*). Adults aged ≥65 years accounted for approximately two thirds of all COVID-19–associated hospitalizations during October 2023–April 2024, with adults aged ≥75 years accounting for approximately one half of hospitalizations and in-hospital deaths. During the 7-month period, cumulative population-based hospitalization rates among all adults aged ≥75 years approached one in 100. These findings suggest that COVID-19–associated hospitalization among adults aged ≥65 years remains a public health concern.

The Advisory Committee on Immunization Practices has updated COVID-19 vaccine recommendations as SARS-CoV-2 has continued to evolve (*4*,*5*). In this analysis, approximately 90% of adults hospitalized during October 2023–April 2024 had not received the recommended 2023–2024 formula dose; approximately one half had not received any COVID-19 vaccine since September 1, 2022, including adults aged ≥65 years. Receipt of COVID-19 vaccine has been demonstrated to reduce the risk for COVID-19–associated hospitalization (*6*).

Disparities in COVID-19–associated hospitalization among adults by race and ethnicity persisted during the study period. Cumulative hospitalization rates among AI/AN and Black adults were 30%–40% higher than were those among Hispanic and White adults. Published data for July 2021–August 2022 showed that cumulative age-adjusted hospitalization rates among adults were approximately twice as high among AI/AN and Black adults as among White adults, and 40% as high compared with Hispanic adults (*7*). These data suggest that racial and ethnic disparities in rates of COVID-19–associated hospitalization among adults continue but might have decreased since July 2021–August 2022. In addition to disparities in rates of COVID-19–associated hospitalization, data from the National Immunization Survey indicate that racial and ethnic disparities among adults exist in COVID-19 vaccination coverage. The percentage of adults who received the 2023–2024 formula dose was highest among White adults relative to all other racial and ethnic groups.*** This disparity in vaccination coverage might contribute to continued racial and ethnic disparities in rates of COVID-19–associated hospitalizations among adults.

Approximately one in six adults hospitalized with COVID-19 was a resident of an LTCF. These findings are consistent with published literature demonstrating high rates of COVID-19–associated hospitalization and low prevalence of COVID-19 vaccination (40.5%) among nursing home residents during October 2023–February 2024 (*8*).

Most adults hospitalized with COVID-19 had two or more underlying medical conditions. A published analysis of COVID-NET data from early in the pandemic found a fourfold increased risk for COVID-19–associated hospitalization among adults with two or more underlying medical conditions, even after adjusting for age, sex, and race and ethnicity (*9*). These data suggest that continued efforts are needed to prevent hospitalizations among adults with multiple underlying conditions.

Vaccination and nonpharmaceutical interventions such as hand hygiene and avoiding exposure to persons with respiratory symptoms can reduce the risk for contracting SARS-CoV-2. In addition, for persons with SARS-CoV-2 infection who are at high risk for progression to severe disease, receipt of early outpatient treatment with ritonavir-boosted nirmatrelvir (Paxlovid, Pfizer), remdesivir (Veklury, Gilead), or molnupiravir (Lagevrio, Merck & Co., Inc.) can reduce the risk for severe outcomes (*10*). Vaccination, other measures to reduce the risk for contracting SARS-CoV-2, and early antiviral treatment are important tools for preventing hospitalization among adults at increased risk for hospitalization, including those aged ≥65 years, residents of LTCFs, and persons with underlying medical conditions.

### Limitations

The findings in this report are subject to at least five limitations. First, COVID-19–associated hospitalizations might have been missed because of hospital testing practices; therefore, hospitalization rates might be underestimated. Second, a patient’s primary complaint at the time of admission is subject to misclassification, potentially resulting in cases being unintentionally included or excluded from this analysis. COVID-19–related illness can still affect the course of hospitalizations even if COVID-19–related illness was not the primary complaint upon admission. Third, vaccination status might be misclassified if immunization information systems data are incomplete; therefore, proportions of vaccinated patients might be underestimated. Fourth, these data only describe in-hospital deaths; deaths among patients discharged to hospice or who died elsewhere after hospitalization are not included. Finally, COVID-NET catchment areas include approximately 10% of the U.S. population; thus, these findings might not be nationally generalizable.

### Implications for Public Health Practice

COVID-19–associated hospitalizations continue to largely affect adults aged ≥65 years. All adults, especially those aged ≥65 years and others at increased risk for progression to severe COVID-19 illness, including residents of LTCFs, should reduce their risk for COVID-19–related hospitalization and severe outcomes by receiving recommended COVID-19 vaccines, adopting measures to reduce risk for contracting SARS-CoV-2, and seeking early outpatient antiviral treatment after receipt of a positive SARS-CoV-2 test result.

## Footnotes

* COVID-19–associated hospitalizations are defined as those among persons who have received a positive SARS-CoV-2 reverse transcription–polymerase chain reaction or rapid antigen detection test result during hospitalization or ≤14 days before admission.

† Selected counties and county equivalents in California, Colorado, Connecticut, Georgia, Maryland, Michigan, Minnesota, New Mexico, New York, Oregon, Tennessee, and Utah for the 2023–2024 surveillance season.

§ Age-adjusted rates were calculated using the direct method using the U.S. Census Bureau Vintage 2022 population estimates.

¶ The COVID-NET surveillance season extends year-round from epidemiologic week 40 through epidemiologic week 39, which roughly aligns with October–September of the following year. To compare with the analytic period in this study, the season was divided into surveillance weeks 40–17 (approximately October–April) and epidemiologic weeks 18–39 (approximately May–September). The 2019–2020 surveillance season began on March 1, 2020; data for that surveillance season are presented as epidemiologic weeks 10–17 and weeks 18–39.

** Vaccination status includes receipt of the 2022–2023 (bivalent) or 2023–2024 formula vaccine. The 2022–2023 formula (bivalent) vaccine was recommended by the Advisory Committee on Immunization Practices on September 1, 2022. The 2023–2024 formula vaccine was recommended on September 1, 2023. Vaccination status was assessed between September 1, 2022, and the date of hospital admission.

†† 45 C.F.R. part 46.102(l)(2), 21 C.F.R. part 56; 42 U.S.C. Sect.241(d); 5 U.S.C. Sect. 552a; 44 U.S.C. Sect. 3501 et seq.

§§ The 1,861 COVID-19–associated hospitalizations among children and adolescents aged ≤17 years represented 4.6% of total COVID-19–associated hospitalizations and are not further described in this analysis.

¶¶ Among the 38,900 COVID-19–associated hospitalizations among adults, data were abstracted from a sample of 1,754. Among these, 84 (4.8% [unweighted]) persons were pregnant, and 350 (19.9% [unweighted]) reported primary complaints upon admission that were not likely related to COVID-19–related illness and were excluded.

*** https://www.cdc.gov/covidvaxview/weekly-dashboard/adult-vaccination-coverage.html (Accessed September 27, 2024).
